# *Strongyloides stercoralis* hyperinfection syndrome mimicking pseudomembranous enteritis, complicated by *Escherichia coli* bacteremia and *Pneumocystis jirovecii* pneumonia in a patient after immunosuppressive therapy: a case report

**DOI:** 10.1186/s12879-022-07670-2

**Published:** 2022-09-24

**Authors:** De-Han Cai, Jun Wang, Xiao-Lin Fang

**Affiliations:** 1 Nephrology Department in Jiangxi Provincial People’s Hospital Affiliated to Nanchang Medical College, NanchangJiangxi, 330006 China; 2 Department II of Respiratory and Critical Care in Jiangxi Provincial People’s Hospital Affiliated to Nanchang Medical College, Aiguo Road 92#, Donghu District, Nanchang, 330006 Jiangxi China

**Keywords:** *Strongyloides stercoralis* hyperinfection syndrome, Pseudomembranous enteritis, *Clostridioides difficile* infection, *Escherichia coli*, *Pneumocystis jirovecii*, Corticosteroids

## Abstract

**Background:**

Strongyloidiasis, caused by *Strongyloides stercoralis* (*S. stercoralis*), is endemic worldwide, especially in countries with warm and humid climates. *Strongyloides stercoralis* hyperinfection syndrome (SHS) is an extremely serious manifestation of strongyloidiasis, which results from an acute exacerbation of auto-infection and is often fatal.

**Case presentation:**

We present a case of SHS mimicking pseudomembranous enteritis with a final definitive diagnosis of a triple infection including *S. stercoralis, Escherchia coli* (*E. coli*) and *Pneumocytis jirovecii* (*P. jirovecii*) that occurred in a microscopic polyangiitis (MPA) patient after immunosuppressive therapy. SHS, together with *E. coli* bacteremia and *Pneumocytis jirovecii* pneumonia (PJP) in the same patient, is rare in clinical practice, which is first reported worldwide, to our knowledge. After the diagnosis was confirmed, the treatment protocol was quickly adjusted; however, the patient’s life could not be saved.

**Conclusion:**

This case reminds us of the necessity to consider strongyloidiasis as a differential diagnosis in immunocompromised populations who live in or have visited to *S. stercoralis* endemic areas, especially patients with suspected pseudomembranous enteritis, even if stool examination, serological tests, and eosinophilia are negative. For this group, it is advisable to complete the relevant endoscopy and/or PCR as soon as possible. The fundamental solution to prevent this catastrophic outcome is to implement effective preventive measures at multiple levels, including physicians, patients, and relevant authorities.

## Background

Strongyloidiasis is one of the most neglected parasitic diseases, because it often manifests as chronic infection with mild and non-specific symptoms [1, 2]. *S. stercoralis* is a soil-transmitted nematode and is the only parasite that can switch back and forth between free-living cycles (rhabditiform larvae) and parasitic cycles (filariform larvae). Rhabditiform larvae are excreted in the stools of infected individuals and can develop into infective filariform larvae in warm, moist soil, which can pierce intact skin and infect a new host, invade the lungs via the bloodstream, migrate to the small intestine to settle, reach maturity, and replicate. *S*. *stercoralis* has two ways of auto-infection: the rhabditiform larvae develop into filariform larvae and then enter the bloodstream from the intestine, or they do not develop into filariform larvae but enter the bloodstream directly from the intestinal mucosa (endo auto-infection); they enter the body from the perianal skin after being excreted in the feces (exo auto-infection) [3]. In this case, we discuss the challenges of confirming the diagnosis of strongyloidiasis and its management in the presence of complex comorbidities.

## Case presentation

The patient was a 66-year-old local farmer who was admitted to Jiangxi Provincial People’s hospital on September 28, 2021, with complaints of abnormal renal function for more than a year, fever and cough for one month. One year ago, he was found to have urine occult blood ++++, urine protein 2084 mg/24 h (normal range < 150 mg/24 h), creatinine 411 µmol/L (normal range 30–110 µmol/L), urea nitrogen 13.8 mmol/L (normal range 3.2–7.1 mmol/L), perinuclear antineutrophil cytoplasmic antibody (p-ANCA) positive, anti-myeloperoxidase antibody (anti-MPO) 209.9 U/mL (normal range 0–20 U/mL. Based upon above indicators and after ruling out other possible secondary vasculitic diseases, we diagnosed the patient with MPA kidney involvement, and treated with methylprednisolone (40 mg per day) for 7 days and cyclophosphamide (0.6 g per month) for 3 months. Subsequently, cyclophosphamide was reduced to 0.8 g every 3 months, and the corticosteroid was tapered to prednisone 10 mg/day. In the previous year the patient was found to have steroidal diabetes mellitus and was started on insulin and gliquidone tablets.

One month before admission, the patient developed chills and fever, with a maximum temperature of 39 °C, after working in a vegetable garden. He also had cough, sputum and chest tightness, mild abdominal pain and diarrhea, 3–5 times/day, but no chest pain or dyspnea.

The results from local Leping City People's Hospital displayed glycated hemoglobin 6.9% (normal range 4–6%), p-ANCA negative, PCT 9.6 ng/mL (normal range < 0.15 ng/mL), and blood culture growth of *E. coli*. Chest computed tomography (CT) showed some reticular fibrous cords in the right lung, and whole abdominal CT showed no significant abnormalities.

On September 28, the patient still had fever, with the temperature fluctuating between 37.3 and 38.2 °C, despite half a month of intravenous administration of moxifloxacin (0.4 g) per day and sulperazone (3.0 g) twice a day. In addition, respiratory symptoms were not alleviated. The patient was transferred to our hospital.

**Fig. 1:**
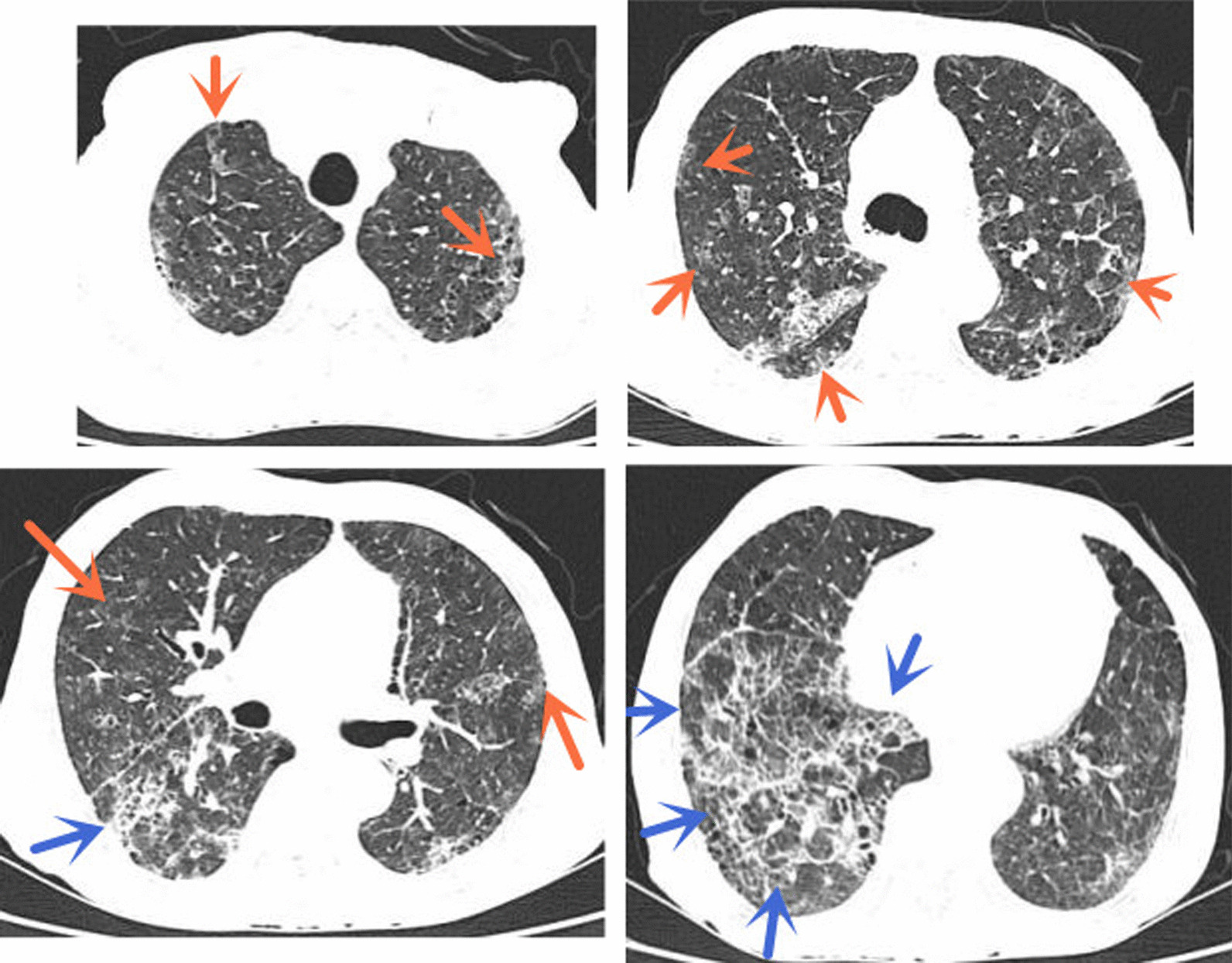
Computed tomography showed diffuse distribution of multiple ground glass opacities (yellow arrow), fibrous cords and reticular shadows (blue arrow) in both lungs (it was done on 28th September 2021)

On admission, blood count showed white cells counts 8.9 × 10^9^/L (normal range 4–10 × 10^9^/L), neutrophils 92.7% (normal range 50–70%), eosinophils 0.03 × 10^9^/L (normal range 0.05–0.5 × 10^9^/L), hemoglobin 83 g/L (normal range 120–165 g/L), platelets 61 × 10^12^/L. Blood biochemistry showed an albumin 18.7 g/L (normal range 35–50 g/L), creatinine 180 µmol/L, urea nitrogen 8.47 mmol/L, LDH 385 U/L (normal range 109–245 U/L), BDG 203.67 pg/mL (normal range < 20 pg/mL). The parasite antibodies (including *Schistosoma, Schistosoma hepatica, Schistosoma lung, Cysticercus, Toxoplasma, Schistosoma mansoni, Hydatid and Angiostrongylus cantonensis*) and HIV antibodies were negative. Blood gas analysis showed pH 7.428, PaCO_2_ 18.2 mmHg, PaO_2_ 77.6 mmHg, Lactic acid of 1.29 mmol/L, and SaO_2_ of 95% (on air). Stool examination did not reveal any occult blood or parasites. A repeat chest CT scan showed more lesions than before (Fig. 1). Abdominal CT revealed thickening of the intestinal wall. We took this patient off coritcosteroids and treated him with meropenem (500 mg, intravenous drip, twice a day) to control *E. coli* bacteremia and nutritional support.

However, on the second day of admission, the patient’s diarrhea and abdominal pain worsened, with yellow watery stools, approximately 10–16 times/day. Stool examination indicates many white blood cells and red blood cells. Multiple stool cultures did not reveal pathogenic bacteria. The patient refused colonoscopy. According to the opinion of the gastroenterologist, the patient had been on broad-spectrum antibiotics for more than half a month, and a clinical diagnosis of pseudomembranous enteritis could be made in terms of the current clinical presentation, even if stool cultures did not reveal *Clostridium difficile* (*C. difficile)*. It was recommended to empirically treat with vancomycin to observe the efficacy, and supplement with medication to regulate intestinal flora. However, the patient did not experience any reduction in abdominal pain and diarrhea after 3 days of treatment with oral vancomycin.

**Fig. 2:**
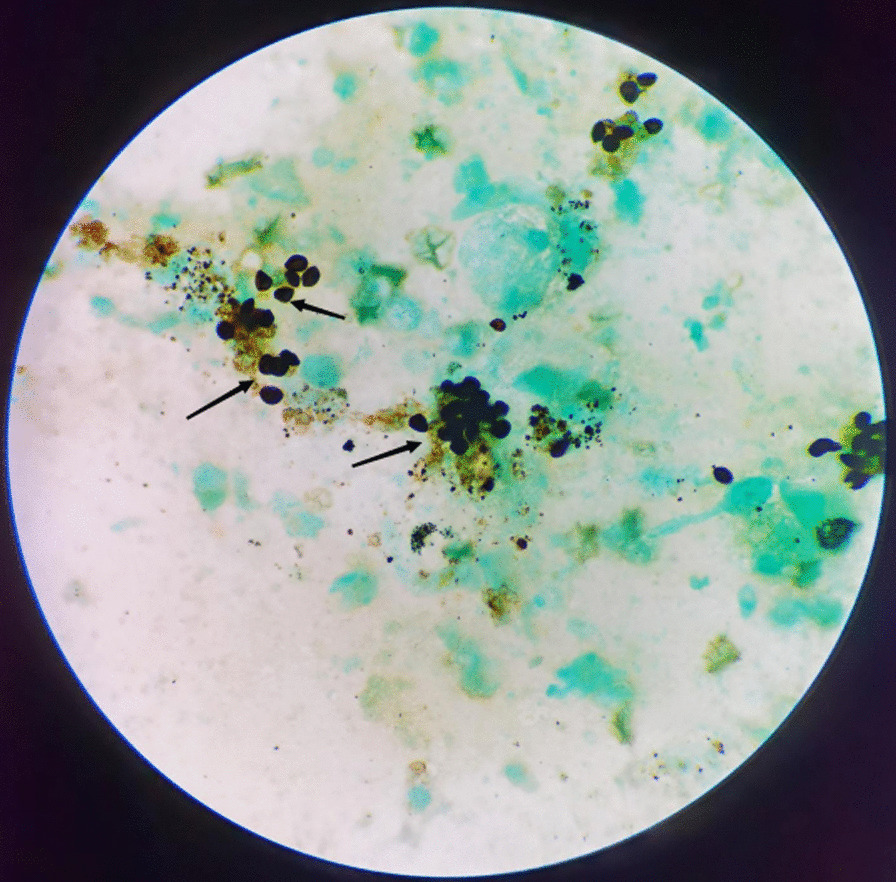
* Pneumocystis jirovecii* were found in BALF. (Black arrow, hexamine-silver staining) Magnification × 1000

**Fig. 3:**
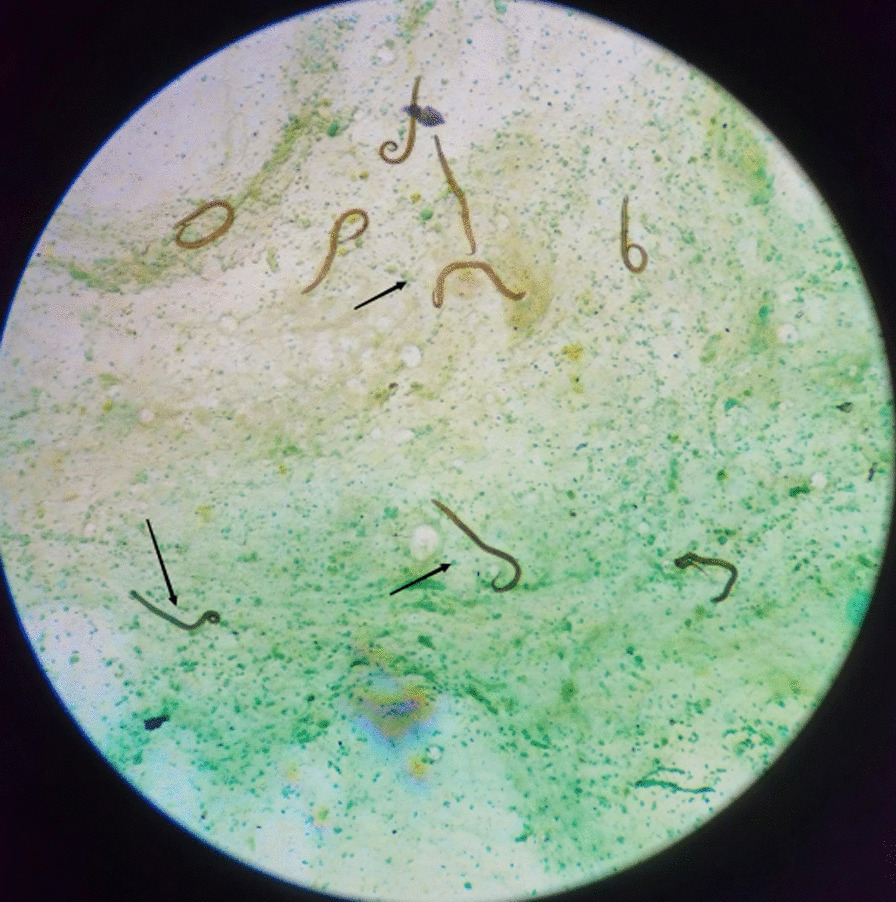
A large number of filariform larvae of *S. stercoralis* were found in BALF. (black arrow, hexamine-silver staining) Magnification × 200

Bronchoscopy was performed, and filariform larvae of *S. stercoralis* as well as *P. jirovecii* were found in BALF on the fifth day after admission (Figs. 2, 3). Therefore, the patient was diagnosed with SHS, PJP and *E. coli* bacteremia. The treatment regimen was adjusted to albendazole (400 mg, orally, twice a day), cotrimoxazole (SMX 0.8/TMP 0.16 g, orally, four times a day) and meropenem (500 mg, intravenous drip, thrice a day) for anti-infection on September 30.

However, two days later, the patient had a high fever, along with marked respiratory distress. Blood gas analysis on October 2 indicated pH 7.39, PaCO_2_ 20.8 mmHg, PaO_2_ 63.8 mmHg, Lactic acid of 4.76 mmol/L, and SaO_2_ of 93% (noninvasive ventilator-assisted ventilation). The patient developed uncorrectable fulminant diarrhea and septic shock, and died 10 days after admission to our hospital.

## Discussion and conclusion

Approximately 100–370 million people are infected *S. stercoralis* worldwide, mainly distributed in tropical and subtropical zones [4]. Infected population tends to come from lower socioeconomic classes, living or working in unhygienic environments, such as farmers, loggers, and miners. Walking barefoot, engaging in activities that require skin contact with soil, and poor sanitation are considered risk factors for infection [5]. Since the parasite can be continuously auto-infecting the host, many patients remain asymptomatic or have mild symptoms for decades, even after leaving the endemic area and going unnoticed [6].

Once the disease occurs in immunodeficient patients, there is a high risk of developing SHS, leading to catastrophic outcomes with mortality rates of up to 80% [7]. The lungs, intestine, and skin are the main organs involved in strongyloidiasis. In hyperinfection syndrome, the typical life cycle is amplified due to the acceleration of auto-infection, allowing for a surge in parasite load [8]. The detection of larvae in respiratory tract specimens is an iconic feature of hyperinfection, as demonstrated in this case [9].

Multiple drug exposures (including some immunosuppressives, antineoplastic agents and biologics) and many diseases (such as human T-lymphotropic virus infection, hypogammaglobulinemia, hematologic malignancies, and organ transplantation) can be pre-disposing factors for SHS, with corticosteroids being the most common underlying cause [10]. Corticosteroids can affect immunity by inhibiting eosinophil proliferation, suppressing the giant cell response, promoting apoptosis of Th-2 lymphocytes, and inducing differentiation of filariform larvae, thereby accelerating auto-infection [8]. It is believed that the occurrence of disease is not related to the dose, duration, or route of corticosteroids administration [10].

In this case, SHS mimicked the manifestations of pseudomembranous enteritis. Both pseudomembranous enteritis and SHS can present with large amounts of yellow watery stools. Routine stools of both may show white blood cells and red blood cells. Both abdominal CT may display thickening of the intestinal wall. Cephalosporins and Fluoroquinolones account for the top two medications in the predisposing antibiotic use to *Clostridioides difficile* infection (CDI) [11]. Enzyme immunoassays for toxins and glutamate dehydrogenase (GDH) for the detection of CDI are not available in our hospital. In the clinical context of a patient with worsening diarrhea and abdominal pain after a longer period of broad-spectrum antibiotics, it is easy for physicians to misdiagnose as pseudomembranous enteritis. However, the patient's condition did not improve after the administration of vancomycin. Instead, the gastrointestinal and respiratory symptoms continued to deteriorate. Multiple stool culture did not find *C*. *difficile*. The diagnosis was not finalized until *S. stercoralis* were found in BALF.

The relationship between strongyloidiasis and *enterobacteriaceae* infections has been well defined. Invasion of the intestinal wall can induce bacterial translocation from the leaky intestinal epithelium, and strongyloidiasis is often associated with *Enterobacteriaceae* infections [12]. However, when a patient presents with an enterobacteriaceae bacteremia first, physicians often only treat it and ignore the real cause that may lie behind it. In this case, the patient already had diarrhea and abdominal pain initially but did not receive attention. When the symptoms worsened, it was misdiagnosed as pseudomembranous enteritis. If physicians had reflected on the source of the *E. coli* bacteremia at the beginning of the patient’s current illness, strongyloidiasis might have been detected earlier, and the patient's outcome could have been different.

In present case, stool examination, eosinophil and parasite antibody tests were negative or normal. Owing to the detection method and intermittent excretion of the parasite, it is not easy to detect the parasite using the direct smear method [13]. The Baermann technique and agar plate culture have higher sensitivity. Multiple stool examination can also increase the positive rate. Eosinophils are common markers of parasitic infections. However, eosinophil elevation is more pronounced in the early stages of the disease and less reliable in immunocompromised hosts [14]. There are data indicating that eosinophilia can be present in 70% of *Strongyloides* infections but only in 20% of patients with SHS [8]. Enzyme Linked Immuno Sorbent Assay (ELISA) has been reported to have 84–95% sensitivity and 82–100% specificity, but is often a false negative result in immunosuppressed individuals [14]. PCR is recommended to be more suitable for confirming the diagnosis than screening [15]. Hence, for immunodeficient group with SHS, relevant endoscopy and/or PCR should be performed to obtain an early basis for *S. stercoralis* infection.

Its greater safety, efficacy, and tolerability make ivermectin the standard treatment for SHS. Ivermectin is available via the oral, rectal, and subcutaneous routes of administration, although only oral administration is currently permitted in humans [16]. As there is no ivermectin in our country, let alone a parenteral route of administration, oral albendazole tablets should be chosen.

The incidence of PJP is increasing in HIV-negative susceptible populations. HIV-positive PJP patients tend to progress sub-acutely, whereas HIV-negative patients often progress acutely within a few days and have a high mortality rate [17]. Corticosteroids are recommended when HIV-positive PJP patients present with hypoxemia (PaO_2_ < 70 mmHg on air) [18]. Nevertheless, it is controversial whether coticosteroids is beneficial in non-HIV PJP patients.

Both *P. jirovecii* and *S. stercoralis* can cause critical pneumonia. In this case, it was difficult to define the proportion of each of the two infections in the development of pneumonia. Both may have multiple ground-glass opacities (GGO) on chest imaging, but the GGO in PJP is often upper lobe dominant or extends peripherally from the perihilar regions. This was not consistent with the imaging findings in this case. The patient had significant interstitial lesions, and imaging of PJP only shows such changes at an advanced stage [19, 20]. Moreover, the patient's LDH and BDG indices were not significantly elevated on review. Therefore, we concluded that the patient’s pneumonia was predominantly caused by SHS, and we did not add corticosteroids. In terms of the overall course of the patient's disease, we believe that SHS was the main cause of death.

Most reported cases of strongyloidiasis ended poorly, even after a clear diagnosis and etiological therapy. In addition to clinicians being vigilant about the disease, prevention is an indispensable aspect. Owing to the use of corticosteroids or other immunosuppressive agents, and in some clinical contexts such as malignancy, organ transplantation, the disease is increasingly exposed incidentally, reminding us that its true prevalence is probably far underestimated. Epidemiological research data on diseases in many regions have not been updated or are even missing. Therefore, it is necessary to screen the population in endemic areas to obtain accurate data on the disease and treat screened patients in a timely manner, rather than waiting for an outbreak to be detected and rushing to respond. It has been pointed out that in high-risk groups, especially those preparing to use corticosteroids, screening for the worm must be performed with at least two tests [21]. Using the proper combination of tests, the positive rate of patients in the mild or early stages of the disease was significantly higher than that of patients with SHS. There are also some radical views that for high-risk groups, it is possible to go directly to anti-parasitic treatment [22]. In less-developed countries or regions, adequate drainage system facilities and regular deworming are required. High-risk groups should have good personal protection, develop good hygiene habits, and protect their feet when in contact with soil at work or in life [23].

In patients with bacteremia, reflection on the origin of the bacteria can assist in identifying possible concealed diseases. It is important to maintain a high level of vigilance against the possibility of strongyloidiasis in immunocompromised populations who live in or have visited to *S. stercoralis* endemic areas, especially those who are suspected of pseudomembranous enteritis. A combination of tests including relevant endoscopy and/or PCR should be conducted to establish a diagnosis as soon as possible in immunodeficient group. Ivermectin is preferred if available, and the appropriate route of administration is chosen according to the conditions. When strongyloidiasis is combined with PJP, corticosteroids therapy should be weighed. Prevention and control measures for strongyloidiasis require the concerted efforts of physicians, patients, and relevant government agencies.

## Data Availability

The data are available from the corresponding author on reasonable request.

## Abbreviations

*S. stercoralis*: *Strongyloides stercoralis*
SHS: *Strongyloides stercoralis* Hyperinfection syndrome
p-ANCA: Perinuclear antineutrophil cytoplasmic antibody
anti-MPO: Anti-myeloperoxidase antibody
MPA: Microscopic polyangiitis
PCT: Procalcitonin
LDH: Lactate dehydrogenase
BDG: 1-3-β-D-Glucan
CT: Computed tomography
Lac: Lactic acid
*E. coli*: *Escherichia coli*
BALF: Bronchoalveolar lavage fluid
CDI: *Clostridioides difficile* Infection
*P. jirovecii*: *Pneumocystis jirovecii*
PJP: *Pneumocystis jirovecii* Pneumonia
HIV: Human immunodeficiency virus
TMP/SMX: Trimethoprim/sulfamethoxazole
PCR: Polymerase chain reaction
